# Language at a glance: How our brains grasp linguistic structure from parallel visual input

**DOI:** 10.1126/sciadv.adr9951

**Published:** 2024-10-23

**Authors:** Jacqueline Fallon, Liina Pylkkänen

**Affiliations:** ^1^ Department of Psychology, New York University, New York, NY 10003, USA.; ^2^ Department of Psychology and Neuroscience, University of Colorado Boulder, Boulder, CO 80309-0345, USA.; ^3^ Department of Linguistics, New York University, New York, NY 10003, USA.

## Abstract

Human brains grasp the gists of visual scenes from a single glance, but to what extent is this possible for language? While we typically think of language in terms of sequential speech, our everyday experience involves numerous rapidly flashing written notifications, which we understand instantly. What do our brains detect in the first few hundred milliseconds after seeing such a stimulus? We flashed short sentences during magnetoencephalography measurement, revealing sentence-sensitive neural activity in left temporal cortex within 130 milliseconds. These signals emerged for subject-verb-object sentences regardless of grammatical or semantic well-formedness, suggesting that at-a-glance language comprehension begins by detecting basic phrase structure, independent of meaning or other grammatical details. Our findings unveil one aspect of how our brains process information rapidly in today’s visually saturated world.

## Teaser

The brain’s magnetic fields reveal ultrarapid detection of syntax in at-a-glance language perception.

## INTRODUCTION

Human language is a multimodal system: We can transmit linguistic expressions from one brain to another via speech, sign, visual writing, braille, and so forth. However, when we study the neurobiology of language, we must always choose some specific way to deliver linguistic input to the participant. This poses a substantial challenge for understanding the neural properties of the amodal system. How can we find the inherent neural properties of the amodal system, uncontaminated by the properties of any specific externalization? An obvious approach is to compare multiple modalities in the same study to find commonalities (*1*–*5*). While important, this is a slow and difficult way to develop a comprehensive understanding of what is shared across modalities. Here, we approach the question of inherent language properties from a different angle, with the specific goal of finding how syntactic and semantic computations are ordered if the input itself does not impose any temporal order. That is, how does the brain order computations if no order is dictated from the input modality? When the brain gets to “decide” when to do what?

Although speech is inherently serial and, therefore, imposes strong constraints for the order in which our brains can process these signals, written language is not. As a static stimulus, the perception of written language could, in principle, proceed very similarly to the perception of a visual scene, whose meaning, or “gist,” can be extracted extremely quickly and accurately (*6*–*8*). Intuitively, we can extract gist-like meanings from full sentences very quickly as well. Imagine, for example, messages flashing on the road or quick notifications on your phone. In augmented reality, this type of rapid reading could become an even more prevalent part of our visual experience.

At the word level, it has long been known that while our brains perceive spoken words phoneme by phoneme, letter perception in reading is highly parallel (*9*, *10*). Thus, although, in speech development, we learn words from strings of phonemes that unfold over time, once we learn to read and the temporal sequencing is no longer necessary, the brain lets go of serial processing and adopts a parallel mechanism instead. This shows that sequential processing of phonemes is not an inherent property of word recognition. Instead, serial processing could arise in language only to the degree dictated by the sensory-motor system that is being used. The mouth is highly limited in its ability to output multiple elements of language at once. Sign language is expressed in a more parallel way, using multiple articulators simultaneously—hands, face, mouth, and the body. Our study addresses how syntactic and semantic computations are neurally organized when the brain is presented with a multiword expression all at once. Will reflections of structural processing emerge slowly, reflecting a cost for having seen multiple words at once? Or do we observe an instant emergence of structure-sensitive processing, with no added time needed because of the parallelism of the input? Consistent with the latter answer, prior research has already shown that the electrophysiological surprisal response N400 is similarly timed for full sentences as for individual words (*11*).

If sentence comprehension can also occur in parallel for visually presented text, then one possible mechanism for rapid at-a-glance comprehension could be quick parallel intake of all the individual words accompanied by their rapid assembly into a sentence. However, recent behavioral studies have shown that our ability for rapid at-a-glance language perception critically depends on the stimulus being a grammatical sentence: For instance, our performance drops substantially if the words of the sentence are scrambled (*12*, *13*). Thus, although parallel presentation imposes no temporal order on the sequence of computations the brain must undertake, linear order of the representational elements clearly matters. The superior perception of grammatical sentences in rapid visual perception has been dubbed the sentence superiority effect (SSE) (*12*). Unpacking the neurobiology of the SSE could give us an insight to the way the language system works if divorced from the seriality of speech and sign. Most prior work on the neurobiology of reading has, however, used unnatural word-by-word presentation of text, as this eliminates artifact-inducing eye movements and controls the time course of processing. Consequently, our understanding of the neurobiology of natural reading remains poor.

While the question of whether a sentence is processed serially even when presented in parallel is important, as it would suggest that serial processing may be an inherent property of the language system, it was not our primary focus in this study. Instead, our main goal was to determine which aspects of syntactic and/or semantic processing are reflected in the earliest neural correlates of sentence superiority. That is, what components of the linguistic representation does the brain “see” first when the stimulus lacks temporal dynamics?

We used spatiotemporally resolved magnetoencephalography (MEG) measurements to determine a neural localization of the SSE and then probed the nature of the underlying computations by manipulating the linguistic properties of the sentences. We used short three-word sentences that are easy to perceive all at once. The sentences were flashed for 300 ms, which is fast enough to eliminate eye movements but long enough to elicit a gist-meaning experience. Our results indicate that the left temporal cortex performs a rough sketch of syntactic structure starting as early as 125 ms after stimulus onset. This is faster than most estimates of even single-word visual perception (*14*), suggesting that the speed arises specifically from the parallel availability of the full sentence, with each word supporting the recognition of the other ones. This allows for rapid matching of the stimulus to top-down knowledge of sentence structure. Just like you can recognize a cup very quickly if you lay your full hand on it, feeling many parts simultaneously (*15*), you are able to understand a sentence very quickly if you lay your eyes on the full sentence all at once.

## RESULTS

### Temporal cortex response to sentences starting at 125 ms

**Fig. 1.**
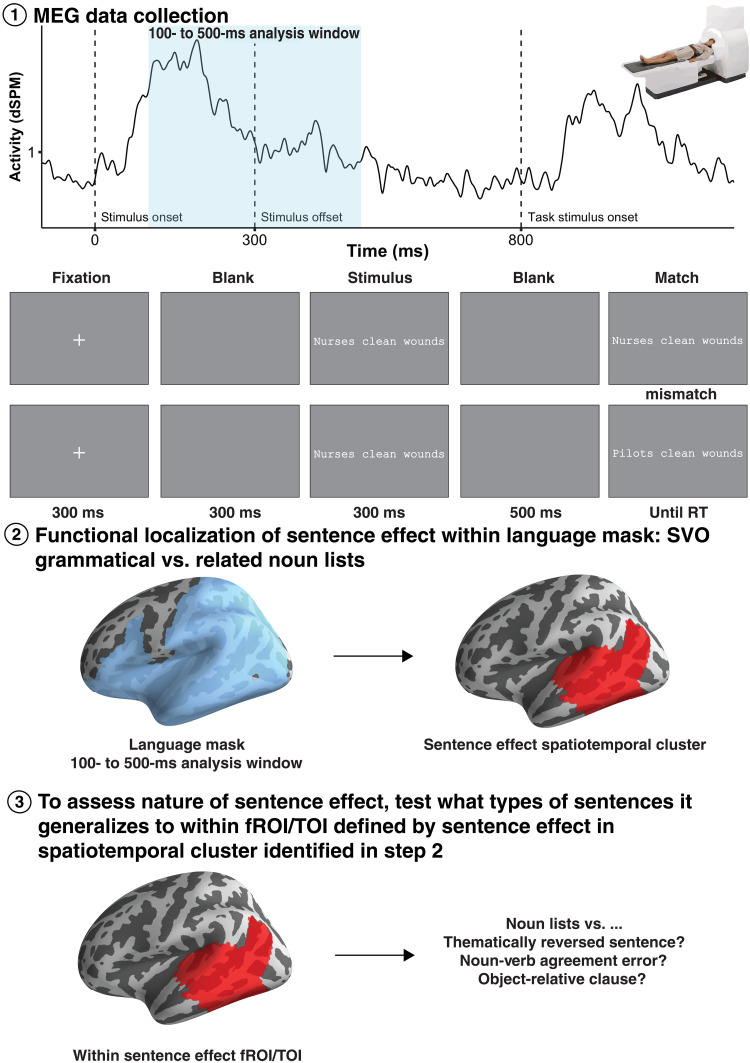
Analysis pipeline for the MEG recordings. (**1**) MEG data were collected continuously during a matching task, in which participants indicated whether two sentences presented right after each other were identical. MEG analysis focused on the responses to the first sentence, which were uncontaminated by decision related signals. (**2**) Source reconstruction of the MEG signals was followed by a spatiotemporal clustering analysis within a broadly defined language mask to isolate signals that showed a neural SSE, that is, elevated signals for our simple SVO sentences as compared to unstructured lists. (**3**) The functional profiles of identified neural SSEs were then queried by assessing whether they replicate for erroneous or complex sentences. RT, reaction time.

Neural activity of 36 native English speakers was measured with MEG, capturing the magnetic fields associated with neuronal currents. Participants saw three-word stimuli for 300 ms, followed by a second stimulus that was either exactly the same as the first stimulus or differed by one word, with a 50% likelihood of same and different trials (Fig. 1). The task was simply to indicate whether the second stimulus matched the first [cf., (*16*)]. The task was intentionally nonsemantic, that is, no understanding of the stimuli was required for performing the task. Therefore, any modulation of response time or neural activity as a function of the grammatical or semantic properties of the stimuli necessarily reflected automatic perception of structure and meaning.

Subject-verb-object (SVO) sentences, such as “nurses clean wounds,” were compared to lists of three semantically related nouns, such as “"hearts lungs livers."” Our aim was to first assess how neural signals to sentences diverge from those elicited by a stimulus that is clearly not a sentence. We then introduced different types of ungrammaticality and complexity to the sentences to elucidate the depth of processing in any observed sentence-sensitive signals. Specifically, we focused on testing the extent to which the fastest neural correlates of sentence detection only operate on fully grammatical sentences or whether they perform shallower detection of structure and/or meaning, insensitive to certain types of syntactic or semantic errors. We also permuted the sentences into syntactically more complex yet grammatical versions, to test whether the first stage of sentence-sensitive signals from the SVO-List contrast are only able to operate for simple sentences or whether they also generalize to more complex structures, which would suggest deeper syntactic processing.

**Fig. 2.**
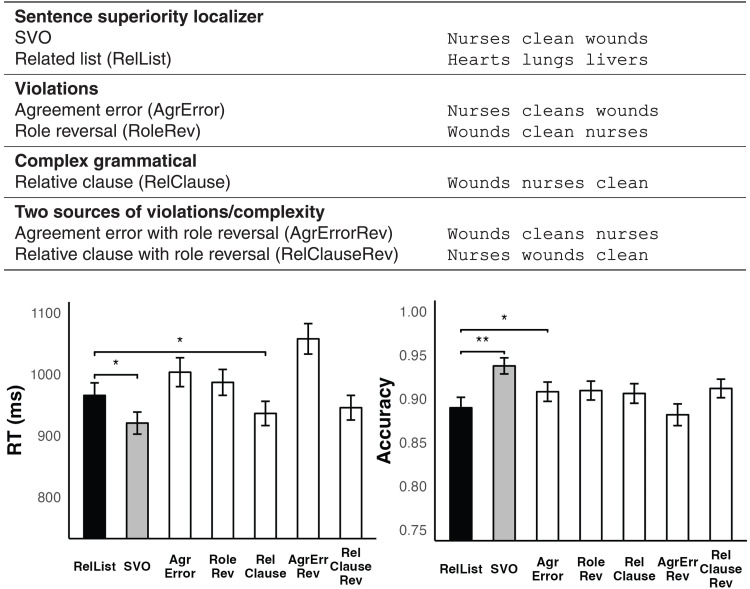
Experiment design and MEG participants’ behavioral results from the matching task. Match responses to grammatical SVO sentences were faster and more accurate than responses to lists of related nouns (RelLists), showing a behavioral SSE. *P ≤ 0.05, **P ≤ 0.01

**Fig. 3.**
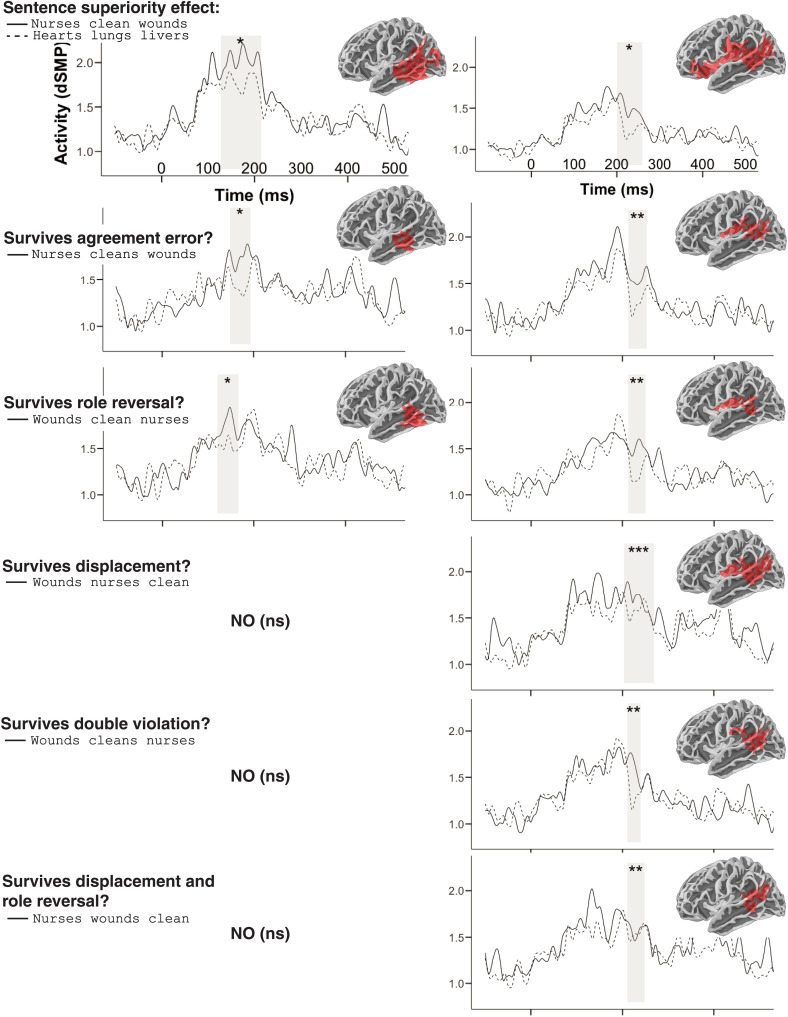
MEG results for the sentence superiority localizer and subsequent tests of its generalizability. Two clusters of elevated neural signals were identified for SVO sentences as compared to the unstructured lists: an early cluster in middle posterior temporal cortex (left) and a slightly later one in ventral frontoparietal cortex. The early cluster replicated for agreement errors and role reversals, but not for relative clauses or the conditions combining two sources of processing difficulty. The latter cluster replicated for all erroneous/complex stimuli, which could be driven by the presence of a verb, rather than structure detection. *P ≤ 0.05, **P ≤ 0.01, and ***P ≤ 0.001. ns, not significant.

The SVO-List contrast elicited a clear behavioral SSE (Fig. 2), with match judgments to sentences being significantly faster [*t*(573) = 2.035, *P* = 0.021] and more accurate than those to list stimuli [*t*(*28*) = 3.154, *P* = 0.0019; SVO: mean = 94.06%, SD = 0.08%; list: mean = 89.74%, SD = 1.06%]. Thus, rather than adding cognitive load, the possibility of perceiving the SVO sequences as well-formed syntactic structures had a clearly facilitative effect, consistent with prior behavioral findings (*12*, *13*). In the neuromagnetic signals, SVO sentences were associated with higher activity than lists in left middle temporal cortex starting already at ~125 ms (Fig. 3). These sentence-related signals manifested as two separate spatiotemporal clusters: a slightly earlier one in posterior middle temporal cortex at 127 to 214 ms (*P* = 0.032) and a later one in superior temporal and inferior parietal cortex at 200 to 259 ms (*P* = 0.036). Both of these clusters were treated as functional regions and time windows of interest (fROIs/TOIs) in our subsequent tests assessing the nature of the computations driving these signals. Their localization conformed well with an increasing body of literature implicating the left posterior temporal cortex as a central neurobiological locus for syntax (*17*–*22*). However, the later cluster replicated for every stimulus type that contained a verb, and, thus, we were unable to rule out the hypothesis that this activity was simply associated with verb processing, not sentence processing (see Supplementary Text). The earlier cluster, henceforth “sentence-sensitive cluster,” did, however, show specificity informative for our research questions and is therefore the focus of our subsequent discussion.

### Robustness of sentence-sensitive signals to errors but not to structural complexity

Next, we assessed whether signals in the early sentence-sensitive cluster engaged even if the sentence was not fully well formed. Research on the processing of serially presented sentences has shown that the detection of syntactic word categories and phrase structure is faster than the processing of agreement relations (*23*). We tested whether our sentence-sensitive signals would be elicited even if the sentence contained an agreement error (“nurses cleans wounds”). Sources within the cluster showed an activity increase as compared to the list controls even for these agreement violations [149 to 193 ms; *P* = 0.05; see (*24*) for converging electroencephalography (EEG) evidence; Fig. 3]. Thus, despite the surface similarity of “nurses cleans wounds” and “hearts lungs livers,” the brain very quickly detected that the first stimulus is more sentence-like than the second. The fact that, at this stage of processing, the brain did not see the agreement error suggests that these sentence-sensitive signals do not participate in the computation of agreement relations. Instead, they could reflect rapid detection of phrase structure or a quick grasp of sentence meaning.

To distinguish between the structural and semantic possibilities, we reversed the order of the subject and the verb in our SVO sentences to yield sentences with implausible meanings such as wounds clean nurses. These role reversals have been studied in a sizeable prior EEG literature (*25*–*28*). We hypothesized that if the early sentence-sensitive signals reflect a quick mapping of the subject, verb, and object to ordered conceptual templates such as agent-action-theme, then the effect should disappear if we destroy this mapping. However, the effect did not disappear (120 to 167 ms; *P* = 0.023), suggesting that a plausible meaning is not what drove the effect. A recent hemodynamic study also found equivalent language cortex responses to anomalous and plausible sentences, suggesting largely syntax-driven processing (*29*). However, our effect did disappear when we fronted the object of the sentences to yield relative clauses such as “wounds nurses clean.” Although this clearly harder structure (*30*–*33*) is a well-formed fragment of English (as in “the wounds nurses clean require careful attention”), it did not “count” as a sentence for the rapid signals at 100 to 200 ms. This suggests that the activity responds to sentence structure as long as the structure follows canonical word order, without displacement.

Last, although agreement errors and semantic reversals activated the rapid sentence-sensitive signals, strings containing both an agreement violation and a reversal did not. Thus, the combination of these errors reduced the sentence likeness of the string sufficiently to eliminate the increase in sentence-related activity. A string containing both a role reversal and relative clause structure (“nurses wounds clean”) also failed to drive the signals, consistent with the fact that even a plausible relative clause failed to do so.

## DISCUSSION

### Precedence of rapid phrase structure detection

In providing the brain with the chance to instantaneously grasp a complete, albeit brief, sentence, our objective was to unveil the fastest-emerging structural processing following stimulus onset. What takes precedence in the brain’s response when the stimulus itself has no temporal structure? Research on the neural processing of serially unfolding sentences has revealed both structural (*23*) and conceptual early stages of processing (*34*). In Friederici’s seminal model of sequential sentence comprehension (*23*), structural processing is initiated at 150 to 200 ms after word onset by the identification of syntactic category, that is, whether the word is a noun, verb, adjective, and so forth. When the brain’s syntactic category prediction is violated, as in “Max’s of,” where the preposition “of” takes the place of an expected noun, a left lateral neural response is elicited at ~200 ms by the offending element (*35*). A series of MEG studies has also revealed that at a similar time, around 200 to 250 ms after word onset, the left anterior temporal lobe computes an elementary conceptual combination, combining the feature sets of the composing words (*34*). Last, in serial word-by-word reading, the posterior temporal cortex shows sensitivity to the number of syntactic compositions at the word at around 200 to 400 ms (*21*, *22*, *36*). Here, we asked what type of processing dominates when the only top-down influence is the person’s grammatical knowledge, as opposed to predictions from a temporal preceding context. Does the brain first see meaning or structure?

Our results point to a structure-dominant first stage of processing. Irrespective of whether the sentence conveyed a plausible meaning, SVO sequences elicited an elevated signal in left posterior temporal cortex, adding to the evidence that this region serves as a central site for syntax (*17*–*20*). This signal peaked extremely rapidly, starting at 127 ms. This suggest that these signals are sensitive to rather shallow form-related cues to structure, perhaps taking advantage of the statistical regularities between syntactic categories and word forms (*37*, *38*). An agreement error did not eliminate the effect, that is, these signals discriminated between ill-formed sentences such as “nurses cleans wounds” and unstructured noun lists such as “hearts lungs livers.” Thus, despite the agreement error, the brain “saw” a verb in the middle position of “nurses cleans wounds”*.* However, when we introduced a so-called syntactic displacement to the structure, moving the object to initial position to create a relative clause (“wounds nurses clean”), the neural sentence related increase was no longer elicited. This suggests that the ultrarapid structure detection may be limited to canonical or frequent word orders.

The hypothesis space for what might count as “canonical” or “frequent” is vast and potentially language dependent. Although in fixed word order languages such as English, there is a relatively straightforward notion of basic word order (SVO in the case of English), and, thus, deviations from this order can be considered “noncanonical,” a similar logic is less applicable to free word order languages such as Finnish or Basque. In these languages, rapid at-a-glance comprehension could be primarily dependent on the correct combination of inflectional markers irrespective of linear order. Another relevant factor could be development: Syntactic structures that emerge the earliest in a child’s brain have the oldest neural traces in the adult brain and could therefore be the most available for rapid detection. All these hypotheses can be tested with the approach outlined here, with replications of the early neural SSE serving as the key diagnostic. Another question for future research is whether the nonsentence status of our relative clause stimuli influenced the early structure-sensitive neural signals. Although these stimuli contained a sentence (the relative clause), it was embedded, and the overall stimulus was a noun phrase. It would be surprising if the nonsentence status, rather than the more complex structure, was the relevant factor, but this hypothesis remains open for further investigation.

Considering our finding in the context of extant models, it resonates with phase 1 in Friederici’s (2002) original sentence processing model (*23*), in which syntactic word category is recognized and basic phrase structure is built. The timing of our effect, 127 to 214 ms, is remarkably similar to the timing of phase 1, posited for 150 to 200 ms. Of course, the unexpected aspect of our result is that it is obtained for a parallel input. Thus, instead of delaying structure detection, the simultaneous availability of all cues, if anything, appears to speed it up. Previous research using serial presentation has shown that rapid structure detection, as described in Friederici’s phase 1, heavily relies on a highly predictive context (*35*). In our study, however, there is no such context; the stimulus is presented all at once, without any syntactic prediction from a preceding context. Thus, surprisingly, it may be that the facilitative effect of having all structural cues available simultaneously is strong enough to override the need for prediction. There is also recent behavioral evidence that lends plausibility to our finding, demonstrating that the reading brain can extract syntactic category information from multiple words with just 50 ms of exposure (*39*). Given this, it is actually not so surprising that, approximately 70 ms later, a rudimentary phrase structure may be detected, as suggested by our results.

Our findings underscore the importance of fine-grained temporal resolution in exploring the interplay between syntactic and semantic processing. The early sentence-sensitive signals, showing a profile of rapid phrase structure detection, lasted only about 100 ms before being immediately replaced by a different pattern in overlapping regions. In addition, if our stimulus manipulation had been more limited—for instance, including only the first three conditions of the design—then the signals observed at ~120 to 220 ms and ~200 to 250 ms would have appeared functionally identical. This emphasizes the need for rich stimulus designs combined with precise temporal resolution to disentangle syntactic and semantic processing, which are highly intertwined because of the compositional nature of linguistic interpretation (*21*, *34*). An important practical benefit of the rapid parallel visual presentation paradigm is its speed: Because stimulus presentation is fast, one can fit a large number of stimuli into a single recording session, making it possible to address a variety of different hypotheses in a short amount of time. The speed combined with our simple matching task also makes this paradigm highly suitable for populations not able to perform long and/or complex experimental tasks.

### Sentence versus scene perception

A visual sentence is in many ways such as a visual scene: Both are complex stimuli containing many meaningful parts, which together make up a more complex meaning.

Here, we found that neural signals to canonically arranged sentences are enhanced as compared to less typical sequences, containing a displacement. Scene perception is also aided when objects are in their typical locations: An airplane in the sky and a lamp above a table elicit sharper neural representations than scenes with atypically placed objects (*40*). Thus, similar to language, scenes have a certain “grammar” (*41*).

Scene perception is thought to proceed in a global-to-local manner, wherein the gist of the scene can be discerned from a single glance at a display lasting as briefly as 20 ms (*6*, *42*). The gist of a scene is perceived more accurately and earlier than individual objects, although object identities also affect gist recognition, suggesting a parallel and interactive process (*43*). The inability for this parallel perception is attested in simultagnosia, a condition in which one cannot perceive multiple objects in parallel (*44*). Thus, we know that the basic ability for rapid parallel meaning extraction exists in our brains. Given this, why would our brains not use it for language? Individuals with simultagnosia are unable to read text, although they, in most cases, can read individual words (*45*). This suggests similar parallel mechanisms for scene and sentence comprehension.

### Parallel presentation as a useful angle into the neurobiology of syntax

Although speech is the most common way to express language, it is possible that the physical limitations of the mouth as an articulator conceal some of the parallel capacity of language. The parallel potential of language is more revealed in sign languages, which use multiple articulators simultaneously—hands, face, mouth, and the body. However, among attested externalizations of language, written text offers the most parallel percept: Within the confines of our visual field, we can grasp a multiword expression from a single glance. In this work, we have begun to carve out the mechanisms by which our brains achieve this. The earliest stage of at-a-glance comprehension appears to be more structure than meaning driven. Although these findings are still tentative, they raise the possibility that parallel presentation may offer a useful angle into the neural basis of syntax, with only our grammatical knowledge as a top-down modulator of processing, rather than a temporally preceding prior context. Although language processing is dynamic, our linguistic knowledge is static. A static stimulus may offer us a more direct window into this knowledge.

## MATERIALS AND METHODS

### Participants

Thirty-six right-handed native speakers of English with normal or corrected-to-normal vision were recruited at New York University to participate in the MEG experiment. Participants provided written informed consent and were compensated for their time. Data from 7 participants were excluded from analysis because of excessive noise, leaving 29 participants’ data in the final MEG analysis (mean age = 20.42 years, SD = 3.46 years; 25 identified as female).

Given that the behavioral component of the experiment was of interest independent of the MEG measurements, we also recruited 30 additional behavioral participants online on Prolific (https://prolific.co) for increased power. These participants were also native speakers of English with normal or corrected-to-normal vision. They provided written informed consent, were compensated for their time, and followed the same experimental procedures as the MEG participants but did not undergo MEG recording (mean age = 36.17 years, SD = 12.95 years; 22 identified as female). The study was approved by the Institutional Review Board ethics committee of New York University (IRB-FY2016-91).

### Design and stimuli

To create our design (Fig. 2), we first built an initial set of 50 SVO sentences and 50 length-matched, semantically related noun lists, designed to elicit a basic SSE. The SVO sentences were then altered in various ways to determine the nature of the processing driving any observed SSEs. First, we created semantic reversals by swapping the subject and object arguments of the initial, semantically plausible SVO sentences (nurses clean wounds → wounds clean nurses). For this to work, all initial SVO sentences needed to be semantically nonreversible, that is, a reversal needed to yield an implausible meaning for each. The initial SVO stimuli also were presented with agreement violations (nurses cleans wounds) and as more complex object relatives such as wounds nurses clean (as one might encounter in a sentence such as the hospital developed a new protocol for the wounds nurses clean to ensure faster healing). Last, we fully crossed the factors meaning (canonical versus reversed) and structure (SVO versus AgreementError versus RelativeClause), yielding both single and double violations. Although lists of semantically related nouns (hearts livers lungs) were used as the control stimulus to identify neural SSEs (given that our sentences involved semantically related words), we also included lists of unrelated nouns (butchers maps pants) as an additional control, as well as pseudo-word lists, in case no neural SSEs were observed with the related lists as the unstructured control. Since this was not the case, the unrelated and pseudo-word list data were not analyzed further. In all, participants viewed 50 sets of nine conditions (six sentence conditions and three list conditions) for a total of 450 stimulus items. Words ranged from 3 to 8 characters in length (nouns: mean = 5.87 characters, SD = 1.27; verbs: mean = 4.5 characters, SD = 0.86), and the sentences and lists ranged from 13 to 23 characters in length (mean = 18.17 characters, SD = 2.03).

Participants’ task was to indicate whether two-word strings presented one after the other were the same [cf., (*16*, *46*)]. Fifty percent of the stimuli were followed by a task sentence identical to the stimulus (match trials), and the remaining 50% were followed by a sentence with one word from the stimulus replaced by a length-matched, semantically plausible word (mismatch trials). Replacement words for mismatch trials appeared with equal frequency in the first, second, and third positions. Our main aim was to assess whether the maximally straightforward yes responses to the matching trials would nevertheless show effects of higher-level linguistic factors such as sentence status of grammatical complexity or violations. Thus, our behavioral analysis focused on the match trials, and the MEG analysis focused on the preceding neural activity elicited by the critical first sentences of those same trials.

### Procedure

After providing informed consent, participants’ head shapes and the locations of five head position marker coils and three anatomical landmarks (nasion and left and right preauricular points) were digitized using a Polhemus FastSCAN system (Polhemus, Vermont, USA). Participants were instructed that they would see sentences flash on the screen, with a second sentence appearing shortly after, and that their task was to indicate whether the second sentence was the same as the first by pressing a button with their left index or middle finger. A short set of example stimuli was presented to participants to familiarize them with the task. They were then instructed to try to keep their eyes centrally fixated throughout the experiment and to read the sentences for their semantic content.

Participants completed the experiment during continuous an MEG recording while lying on a bed inside a magnetically shielded room. Sentences were projected onto a screen approximately 44 cm away from the participant’s eye in white 20-point monospaced font on a gray background. Each stimulus subtended a horizontal visual angle between 4.7° and 8.4° (mean = 6.064°). Trials began with a 300-ms presentation of a central fixation cross, followed by 300 ms of a blank screen. The stimulus would then appear for 300 ms, followed by 500 ms of a blank screen before the task item would appear and stay on the screen until response. Following the participant’s response, the interstimulus interval was jittered between 600 and 750 ms. The order of trials was randomized for each participant and presented in six blocks of 75 trials with self-timed breaks throughout. The recording session lasted approximately 45 min.

### Data collection and preprocessing

MEG data were acquired on a 160-channel Kanazawa Institute of Technology system (Eagle Technology, Japan) at a sampling rate of 1000 Hz, with an online band-pass filter of 1 to 200 Hz. Magnetic coils were used to record participants’ head positions at the beginning and end of the recording session. MEG data were cleaned of environmental noise using the continuously adjusted least-squares method (*47*) in the MEG160 software (Yokogawa Electrical Corporation and Eagle Technology Corporation, Tokyo, Japan). All subsequent preprocessing and analyses were conducted using the MNE-Python (v. 0.37.6) (*48*) and Eelbrain (v. 0.20.8) (*49*) packages in Python. The data were low-pass filtered offline at 40 Hz, and a common set of six excessively noisy or flatlined channels was removed from all recordings and interpolated using data from surrounding undamaged sensors. Each participant’s data were then visually inspected for any additional bad channels, which were interpolated if present (mean = 1.14 additional bad channels identified). Independent component analysis was then used to identify and remove well-characterized biological and environmental noise artifacts such as heartbeat, blinks, and bodily movement. The data were segmented into epochs spanning −100 to 800 ms relative to the onset of the critical stimulus, that is, the first sentence in the trials. Trials with responses faster than 200 ms and slower than 6 s were first removed to eliminate accidental responses and trials where participants were inattentive or sleepy. From the remaining data, trials with a response time greater than three SDs from the item or participant mean were also removed (mean = 39.6, SD = 4.44). Trials with incorrect responses were excluded (mean = 69.6, SD = 35.53). Last, epochs were rejected if their magnitude exceeded 3000 fT (mean = 2.27, SD = 3.15). On average, 15% of trials were removed based on accuracy, 8% on response time, and 0.04% for noise. Evoked responses were created by averaging epochs within each condition for each participant. The FreeSurfer average brain (*50*) was scaled to fit each participant’s head shape and aligned on the nasion and preauricular points, resulting in a 2562-vertex source space per hemisphere. A forward solution was computed using the boundary element model. The covariance of channel noise was computed using the 100-ms prestimulus interval, which was baseline-corrected separately from the nonbaseline-corrected data used for analysis. The forward solution and covariance were then used to estimate the inverse solution per participant and per condition, assuming a signal-to-noise ratio value of 3, resulting in noise-normalized dynamic statistical parameter maps (dSPM) (*51*). Given our use of an average brain template rather than individual magnetic resonance imagings, our localization results should be interpreted with some caution.

### Data analysis

#### Behavioral data

Behavioral data were analyzed from the same set of participants included in the MEG data analysis, and all Prolific participants, yielding Ns of 29 and 30, respectively, for a total of 59. Trials with reaction times shorter than 200 ms and longer than 6 s were excluded from the analysis, as in the MEG analysis, and then trials with a response time greater than three SDs from the item or participant mean were also removed. Our aim was to focus on the most straightforward measurements, and, thus, we only analyzed the “match” trials, that is, our behavioral results simply reflect participants’ speed and accuracy for indicating that two stimuli are exactly the same. The basic SSE was assessed by a pair-wise comparison of the canonical SVO sentences and the related lists, and then the sentence stimuli were also submitted to a 2 × 3 repeated-measures analysis of variance (ANOVA) with the factors meaning (canonical and reversed) and structure (SVO-gramm, AgreementError, and RelativeClause), to assess the impact of the full manipulation on behavioral responses.

#### MEG data: Isolation of sentence effects within a language mask and testing their sensitivity to violations and complexity

In our MEG data analysis, we first aimed to provide a neural characterization of the SSE (*12*) by identifying neural signals that showed elevated amplitudes for the canonical SVO sentences in comparison to our unstructured yet semantically related list controls, consistent with prior work showing elevated MEG signals in the presence of combinatory language processing (*34*). These increases were hypothesized to reflect the detection of sentence structure. We searched for these signals within a broadly defined language mask including the entirety of the left temporal and parietal lobes as defined by the PALS_B12_Lobes atlas (*52*), as well as the left anterior portion of the occipital lobe (Brodmann area 19), and ventromedial and prefrontal areas (Brodmann areas 10, 11, 44, 45, and 47). The search was performed from 100 to 500 ms after the onset of the first stimulus in our trials, as shown in Fig. 1, motivated by our intent to investigate the initial stages of sentence processing in rapid parallel visual presentation. Since data from the list condition were used both for the neural SSE localizer contrasting canonical SVO sentences and lists, as well as for the subsequent tests assessing the generalizability of those effects to cases involving violations or structural complexity, we used the list-mismatch trials for the functional SSE localizer and the list-match trials for the subsequent tests. Since the task stimulus that determined matches and mismatches was the second stimulus and the MEG signals used in the analyses came from the first stimulus, the analyzed neural data did not reflect components of the match judgments, only automatic processing of the first stimulus. Other than the list trials of the functional localizer, all analyzed MEG data came from the match trials, corresponding to the data used for the behavioral analysis.

Significant differences between the SVO sentences and related noun lists were assessed with a spatiotemporal cluster–based permutation test (*53*), identifying spatiotemporal clusters of at least 10 contiguous sources and 20 ms of duration in which an uncorrected significance of <0.05 was observed in a paired *t* test. For each identified cluster, a test statistic was constructed equaling the summed *t* values of the point-by-point test statistics over the entire cluster. The observed data were then permuted 10,000 times by randomly assigning condition labels within each participant’s data with the final corrected *P* value equaling the ratio of permutations yielding a test statistic greater than the actual observed test statistic (alpha *P* ≤ 0.05).

Having identified sentence-sensitive neural signals, we then proceeded to probe what characteristics of the sentences drove those signals. To do this, we treated the results of the first analysis as a fROI/TOI for subsequent comparisons, in which the grammatical SVO sentences were replaced by versions of those sentences containing violations or complexity, as described above in design and stimuli. MEG responses to these altered stimuli were compared to the so far held-out half of the related list data within the fROI/TOI using the same statistical procedure as in step 1. If the altered sentence replicated the effect observed at step 1, then this was taken as evidence that the sentence-sensitive neural SSE did not detect the anomaly or complexity of the alteration. In reverse, if there was no replication, then this was taken as evidence that the signals generating the neural SSE did participate in the detection of the given anomaly of complexity.
