# Supplementary material for: Language at a glance: How our brains grasp linguistic structure from parallel visual input
Source: Sci Adv. 2024 Oct 23;10(43):eadr9951. doi: 10.1126/sciadv.adr9951 (PMC11498217; doi:10.1126/sciadv.adr9951)
Supplement: Supplementary file 1 — Supplementary Text [file sciadv.adr9951_sm.pdf]

Science Advances
AAAS

Supplementary Materials for

Language at a glance: How our brains grasp linguistic structure from parallel visual input

Jacqueline Fallon and Liina Pylkkänen

Corresponding author: Liina Pylkkänen, liina.pylkkanen@nyu.edu

Sci. Adv. **10**, eadr9951 (2024)
DOI: 10.1126/sciadv.adr9951

This PDF file includes:

Supplementary Text

SUPPLEMENTARY MATERIALS

SUPPLEMENTARY TEXT

Behavioral Results

Our matching task was meant to be easy and the overall task performance reflected that: mean accuracy was 90.6% (SD = 5.7%) for the MEG-only participants (N = 29), and 90.04% (SD = 6.66%) for larger MEG + Prolific sample. Below, we report the behavioral results separately for these two groups, to allow direct comparison of the MEG-only group's behavior to their corresponding MEG data.

MEG-only. In reaction time, a behavioral SSE was observed for the basic contrast between our related noun Lists and the canonical SVO sentences, with the sentences eliciting a ~50ms advantage compared to the lists ($t(573) = 2.035$, $p = 0.021$). Of the deviant/complex test sentences, the grammatical relative clauses replicated the SSE ($t(571) = 1.94$, $p = 0.028$) but none of the other conditions did. This shows that (a) the behavioral SSE was elicited even in the presence of syntactic complexity as in the Relative Clause and (b) the behavioral SSE dropped off as soon as the stimulus had either an agreement error or an implausible role reversal. The 2 x 3 repeated measures ANOVA within the sentence conditions assessing the effects of structure (SVO, Agreement Error, Relative) and meaning (canonical, reversed) showed reliable main effects of both (Structure $F(3,28) = 6.688$, $p = 0.0015$; Meaning $F(1, 28) = 8.379$, $p = 0.00728$). The structure effect was driven by longer reaction times to the agreement violations than to SVOs ($t(28) = 2.79$, $p = 0.0094$)) or relative clauses ($t(28) = 3.4$, $p = 0.002$)) and the meaning effect by longer reaction times to the reversed than the canonical word orders ($t(86) = 2.81$, $p = 0.003$). Structure and meaning did not interact.

Accuracy data for the MEG-only group also showed an SSE in the pairwise comparison between canonical SVOs and the related noun lists ($t(28) = 3.15$, $p = 0.00192$). Of the deviant/complex test sentences, only the agreement violations with canonical thematic role assignments replicated the accuracy SSE ($t(28) = 1.76$, $p = 0.044$). A 2 x 3 repeated-measures ANOVA revealed a marginal main effect of meaning ($F(1,28) = 3.88$, $p = 0.058$), with canonical word orders being more accurate than reversed. There was no significant main effect of structure on accuracy ($F(3,28) = 2.293$, $p = 0.11$).

MEG-only + Prolific. When the Prolific subjects were added to the analysis (n = 30 prolific subjects, n = 59 combined), the pairwise comparison between grammatical SVO sentences and the related lists remained significant both in speed ($t(1225) = 4.09$, $p = 0.000024$) and accuracy ($t(58) = 3.53$, $p = 0.0004$). The 2 x 3 reaction time ANOVA on the sentence stimuli also replicated the main effect structure ($F(3,58) = 11.66$, $p < 0.0001$) and meaning $F(1,58) = 17.82$, $p < 0.0001$) with a highly similar pattern to the MEG-only group. The interaction between Structure and Meaning began to emerge slightly ($F(2,58) = 2.47$, $p = 0.091$), driven by a larger effect of reversal on reaction time for Agreement errors than for the SVO or relative clause stimuli.

Of the deviant/complex test sentences from the combined MEG + Prolific sample, the accuracy SSE replicated for both types of relative clauses (relative canonical: $t(58) =$

1.50, $p = 0.016$; relative reversed: $t(58) = 2.37$, $p = 0.011$). Role reversals were also more accurate than Related Lists (RoleRev: $t(58) = 2.20$, $p = 0.016$). Agreement violations with canonical thematic roles approached a significant accuracy advantage over lists, but agreement violations with reversed roles did not (AgrError canonical: $t(58) = 1.50$, $p = 0.069$; AgrErr reversed: $t(58) = 0.069$, $p = 0.47$).The 2 x 3 accuracy ANOVA on the combined accuracy data revealed significant main effects of structure ($F(3,58) = 7.219$, $p = 0.00111$), but not meaning ($F(1,58) = 2.948$, $p = 0.0913$). The structure effect was again driven by lower accuracy for agreement violations than SVOs ($t(58) = 5.02$, $p < 0.0001$) or relative clauses ($t(58) = 2.54$, $p = 0.0138$).

MEG results

Functional localization of neural Sentence Superiority Effects within a language mask

As shown in Figure 3, our spatiotemporal cluster analysis identified two clusters of increased source-localized MEG activity for canonical SVO sentences over semantically related lists. The first cluster was observed very early at 127-214ms in the left posterior temporal cortex ($p = 0.032$) and the second slightly later at 200-259 ms in left superior temporal, inferior parietal and inferior frontal cortices at ($p = 0.011$). These two clusters served as localizations of the neural Sentence Superiority Effect (SSE), whose underlying functions were then probed by testing the replicability of the neural SSEs for stimuli that were less canonical as sentences, either due to ill-formedness or structural complexity.

Replicability of the neural Sentence Superiority Effects for less sentence-like stimuli

We assumed that if a neural SSE is observed for an ill-formed sentence, this indicates that the neural signals generating the SSE do not detect the ill-formedness but rather treat the stimulus as a “sentence.” In other words, the ill-formed features are not part of the computation underlying that neural SSE. Following this logic, agreement violations and role reversals with implausible meanings were compared to the previously held out half of the related List data within the two fROI/TOIs isolated by the functional localizer. Other than using the fROI/TOI as the search area for the significant clusters, the parameters of this second stage of analyses were the same as those for the functional localizer.

For the early posterior temporal SSE cluster (127-214ms), we observed a replication of the neural SSE both for agreement violations (149-193ms, $p = .05$) and for implausible role reversals (120-167ms, $p = .023$), suggesting that this activity detected aspects of sentence structure other than agreement or plausible meaning. However, the effect did not replicate for the relative clauses, showing that these very rapid neural signals did not “see” a well-formed structure when presented with a grammatical stimulus containing a displaced argument (*wounds nurses clean*). The effect also failed to replicate for both conditions containing two types of ill-formedness/complexity, that is, relative clauses with an implausible reversal and agreement violations with an implausible reversal, suggesting these stimuli were not sufficiently sentence-like for these early signals to rise.

The slightly later, more anterior and more superior cluster (200-259ms) had a different behavior. In fact, it replicated for all our violation/complexity conditions as compared to the related lists (AgreementError 210-253ms, p = .003; Reversal 213-250ms, p = .008; RelativeClause 203-269ms, p = .0001; Relative with reversal 210-249ms, p = .0085; and AgreementError with reversal 210-239ms, p = .0047ms). Although robust, this pattern unfortunately did not speak to our research questions, since the pattern can be explained simply as an effect of the presence of a verb in all the different sentence variants as opposed to the related noun list. The logic of our design depended on at least one sentence condition failing to replicate a neural SSE, but this later cluster did not meet this criterion. Nevertheless, it does reveal a robust effect of some higher level linguistic factor in very rapid evoked activity for multiword parallel expressions, meriting future research to uncover its underlying function.
